# Protein Distribution and Muscle-Related Outcomes: Does the Evidence Support the Concept?

**DOI:** 10.3390/nu12051441

**Published:** 2020-05-16

**Authors:** Joshua L. Hudson, Robert E. Bergia, Wayne W. Campbell

**Affiliations:** 1 Department of Nutrition Science, Purdue University, 700 W State St, West Lafayette, IN 47907, USA; RBergia@purdue.edu (R.E.B.III); CampbellW@purdue.edu (W.W.C.); 2 Department of Pediatrics, University of Arkansas for Medical Sciences, 4301 W Markham St, Little Rock, AR 72205, USA; 3 Arkansas Children’s Nutrition Center, 15 Children’s Way, Little Rock, AR 72202, USA

**Keywords:** protein patterning, muscle mass, fat-free mass, weight loss, higher-protein diet, older adults, aging

## Abstract

There is a shift in thinking about dietary protein requirements from daily requirements to individual meal requirements. Per meal, stimulation of muscle protein synthesis has a saturable dose relationship with the quantity of dietary protein consumed. Protein intake above the saturable dose does not further contribute to the synthetic response; the “excess” amino acids are predominantly oxidized. Given that daily dietary protein intake is finite, finding protein distribution patterns that both reduce amino acid oxidation and maximize their contribution towards protein synthesis (in theory improving net balance) could be “optimal” and is of practical scientific interest to promote beneficial changes in skeletal muscle-related outcomes. This article reviews both observational and randomized controlled trial research on the protein distribution concept. The current evidence on the efficacy of consuming an “optimal” protein distribution to favorably influence skeletal muscle-related changes is limited and inconsistent. The effect of protein distribution cannot be sufficiently disentangled from the effect of protein quantity. Consuming a more balanced protein distribution may be a practical way for adults with marginal or inadequate protein intakes (<0.80 g·kg^−1^·d^−1^) to achieve a moderately higher total protein intake. However, for adults already consuming 0.8–1.3 g·kg^−1^·d^−1^, the preponderance of evidence supports that consuming at least one meal that contains sufficient protein quantity to maximally stimulate muscle protein synthesis, independent of daily distribution, is helpful to promote skeletal muscle health.

## 1. Introduction

In both the United States and Canada, dietary protein recommendations are expressed on a daily basis. For apparently healthy adult men and women, 0.66 and 0.80 g·kg^−1^·d^−1^ of “good-quality” protein denote the protein estimated average requirement and recommended dietary allowance, respectively [1]. These rounded estimates, based on a meta-analysis of 19 nitrogen balance studies, were determined by interpolating the quantity of dietary protein needed per day to achieve zero balance (whole-body nitrogen equilibrium between input and output). When the body is in zero nitrogen balance, protein breakdown presumably equals protein synthesis [2]. By the mid-20th century, it was understood that protein synthesis fluctuated in response to essential amino acid consumption [3,4,5,6]. Consequently, the meal-to-meal pattern of protein intake—the within-day protein distribution—was hypothesized to influence daily protein synthesis, protein equilibrium, and nitrogen balance [7].

With the advent of stable-isotope amino acid methodologies, essential amino acid flux (rate of appearance and rate of oxidation) could be used to measure the effect of consuming protein-containing meals on whole-body protein turnover rates. Net protein balance, the gain or loss of body protein, is partly determined by the natural interplay of amino acid metabolism between the “anabolic” postprandial state and the “catabolic” postabsorptive state [8]. However, net protein balance is more easily addressable at the whole-body level and is an infrequently used tissue-specific (e.g., muscle) technique. In the muscle, a more positive muscle protein net balance is posited to occur primarily through stimulating muscle protein synthesis (MPS) via dietary protein and amino acid consumption. This postprandial anabolic response, however, has a “saturable dose limit”: MPS will not be further augmented by the provision of additional amino acids [9,10]. The amino acids ingested in excess of the saturable dose may not be necessarily “wasted.” Instead, they may suppress protein breakdown to promote a more positive net balance [11,12]; however, while this has been demonstrated on a whole-body level, it remains an untested hypothesis in the muscle. A more positive net balance, either meal to meal or daily, should be beneficial for the maintenance, accretion, or retention of skeletal muscle.

**Figure 1:**
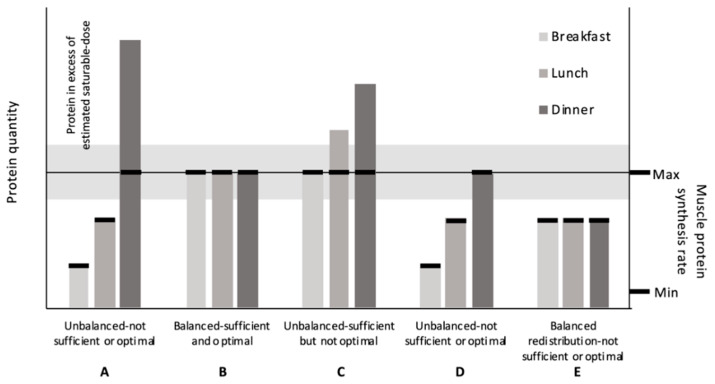
Theoretical relationship between protein quantity within meals and muscle protein synthesis (MPS) rates. (**A**) An example of an unbalanced protein distribution observed in the US. Exceeds the saturable dose limit for protein at one meal. (**B**) An “optimal” protein distribution; 1. provides sufficient protein per meal to maximize MPS without 2. exceeding the saturable dose estimates. (**C**) An unbalanced, higher-protein diet that provides sufficient protein per meal to maximize MPS but greatly exceeds saturable dose estimates at two meals. (**D**) An unbalanced, lower-protein diet that provides only one meal with sufficient protein to maximize MPS. (**E**) A redistributed balanced lower-protein diet; this results in three meals with insufficient protein to maximize MPS. (expanded adaption from Paddon-Jones and Rassmussen Curr Opin Clin Nutr Metab Care 2009, 12: 86–90).

Defining the saturable dose limit provided the conceptual rationale for an “optimal” within-day protein distribution. In general, the within-day protein distribution refers to the relationship between the quantities of dietary protein consumed meal to meal to the overall intake. The relative similarity or dissimilarity of the protein quantities between meals is a criterion we define broadly as being either balanced or unbalanced, respectively. A “perfectly” balanced distribution, regardless of the number of eating occasions, would theoretically result in each meal containing equivalent protein quantities. Conversely, the relative protein quantities between meals should vary considerably in an unbalanced distribution. As one example, an unbalanced distribution has been characterized in the diets of younger and older adults in many Westernized societies as being skewed towards the evening meal (Figure 1A) [13,14,15,16,17,18,19,20,21]. The overwhelming majority of individuals consume 2–4 meals for their primary source of dietary protein; this is opposed to, at the extremes of eating frequency, consuming protein in either a continuous fashion (parenteral or enteral nutrition) or in a single eating occasion. Therefore, we consider this typical eating pattern (2–4 meals) as the status quo for the distribution concept, i.e., the base rate is represented by an unbalanced distribution over a few primary meals. A within-day protein distribution is hypothesized to be “optimal” if the following criteria are met: the protein quantity within each meal (1) meets and (2) does not greatly exceed the physiological saturable dose limit (Figure 1B). This would maximize the utilization efficiency of amino acids shunted away from oxidation towards MPS.

Based on the first principal, each meal should contain sufficient protein to elicit a maximal MPS response. Based on the second principal, an “optimal” protein distribution should avoid either excessive or ecologically unattainable protein quantities that shunt excess amino acids towards oxidation. At “higher” total daily protein intakes, an unbalanced protein distribution could meet and exceed the saturable dose limit at each meal (Figure 1C). However, total protein intake is finite. Consequently, there is an economy to how much protein an individual should, could, or would feasibly consume in a day. Knowing this, we need to work within these bounds when making recommendations. For instance, simply recommending higher total protein intakes seems less ecologically feasible than recommending a redistribution of protein from larger protein-containing meals to lower ones; this would create a more balanced within-day protein distribution. However, if total protein intakes are “low”, simply redistributing dietary protein from an unbalanced (Figure 1D) to a more balanced distribution does not necessarily result in an “optimal” protein distribution (Figure 1E). In fact, a low total protein intake, but balanced protein distribution, may be less sufficient to support skeletal muscle health than a low total protein intake with an unbalanced distribution.

This narrative will review the available (to the authors) research related to the protein distribution concept on muscle-related outcomes. Specifically, we review the evidence behind the claims that a protein distribution pattern other than the base rate—an unbalanced distribution (Figure 1A)—will differentially affect muscle related outcomes. In general, these claims favor the consumption of a balanced protein distribution pattern over the status quo (e.g., “optimal”; Figure 1B): A typical unbalanced protein distribution in which incidentally at least one meal/day contains sufficient protein quantity to maximally stimulate MPS, independent of distribution.

## 2. Observational Research

Observational research is useful for documenting the potential relationship between dietary patterns and clinically relevant endpoints. To date, we are aware of 12 observational studies that investigated associations between the within-day protein distribution and various skeletal muscle-related endpoints [22,23,24,25,26,27,28,29,30,31,32,33] (Table 1). This section has been divided into three subsections based on how the authors of the included studies decided to investigate the protein distribution concept: (1) based on the degree of protein distribution using a coefficient of variation (CV) for protein intake among the main meals; a lower CV denotes a relatively more balanced distribution than a higher CV; (2) based on the number of meals that meet a certain criterion, or threshold, of protein quantity; more meals reaching the target threshold would indicate a “move” towards a more balanced protein distribution; (3) based on when at least a single meal or more does not reach the target threshold; this would indicate a “move” away from a balanced protein distribution.

### 2.1. Degree of Protein Distribution

Prior to the acute randomized controlled trials that popularized the protein distribution concept known today [34,35,36], there was evidence that the within-day protein distribution could influence whole-body nitrogen metabolism [37,38,39]—the details of which will be discussed in a later section. With this early framework, Bollwein et al. [22] stratified participants according to frailty status: non-frail, pre-frail, and frail. For each group, they calculated an average CV among the main meals. Although total daily protein intakes were comparable (~1.1 g·kg^−1^·d^−1^), non-frail participants reported a more balanced protein distribution [0.68 au (0.15–1.24); median (min–max)] than both pre-frail [0.74 (0.07–1.29)] and frail [0.76 (0.18–1.33)] participants. After the retrospective analysis estimating the saturable dose limit in adults was published [10], Gingrich et al. [23] followed up with an investigation into how CV related with muscle mass, strength, and power; however, they reported that no such associations were present in their sample. These results apparently disagree with Bollwein et al. [22]; however, the cohort assessed by Gingrich et al. [23] is more homogenous and apparently “healthier”. Gingrich et al. [23] reported a mean CV of 0.53 ± 0.19 (arbitrary units ± SD) among all participants, which, when compared to the sample from Bollwein et al. [22], is more similar to the median CV of the non-frail volunteers [0.68 (0.15–1.24)] than the other groups. The authors [23] speculated that it is possible that within a cohort of non-frail volunteers with an already relatively balanced protein distribution [22], a more balanced protein distribution would not potentiate an association with muscle strength. These limited results would also suggest that protein distribution may be associated with skeletal muscle-related outcomes in functionally limited older adults but not necessarily in those who are apparently healthy, who tend to be those who are physically active.

Physical activity improves muscle strength in adults and “sensitizes” the muscle to protein feeding, enhancing MPS for up to 48 h after exercise [40]. In light of the lack of published research relating protein distribution to physical function, Ten Haaf et al. [24] combined data from two published studies in the Netherlands. Participants were divided into tertiles based on their protein distribution CV of the main meals: balanced (CV < 0.43); intermediate (CV 0.43–0.62); unbalanced (CV > 0.62). They reported [24] that a balanced protein distribution was associated with greater gait speed compared to the intermediate group only, but not hand grip strength, balance, chair rise ability time, or any of the quality of life outcomes. The adults comprising the total sample produced a wide range of physical activity levels. Within a subgroup of the less physically active adults, who could feasibly be more functionally limited, it is possible that protein distribution would be related with more of the outcomes assessed. This would be consistent with the results from Bollwein et al. [22] and Gingrich et al. [23]; however, this is only conjecture.

To our knowledge, two retrospective cohort studies have been published documenting relationships between protein distribution (CV) and changes in lean mass and physical function. Using baseline and 2 y follow-up data from the Quebec Longitudinal Study on Nutrition as a Determinant of Successful Aging study [25,26], Farsijani et al. [25] investigated whether CV was associated with whole-body and appendicular lean mass either at each time point or with their decline over two years. At baseline, protein distribution was associated with greater lean mass and appendicular lean mass but was not associated with the decline in lean mass over time. Using data from the same parent study, Farsijani et al. [26] performed a similar analysis on muscle strength and mobility outcomes after a three-year follow up. A more balanced protein distribution (CV) was associated with higher muscle-strength scores in men and women and greater mobility scores, but only in men without adjusting for covariates. Protein distribution was not associated with three-year declines in strength and mobility. The authors posited the lack of an association over time may be due to the duration of the follow-up periods. It is possible that two or three years is not long enough to detect associations between protein distribution and changes in lean mass and muscle strength among a free-living cohort. However, this would indicate that if protein distribution does in fact influence muscle-related outcomes, the effect is both small and incremental; it would require individuals to repeat the dietary behavior for long durations in order to marginally benefit.

Collectively, four [22,24,25,26] of five [22,23,24,25,26] studies reported a cross-sectional association between a more balanced protein distribution (CV) and at least one outcome among the cluster of outcomes listed in each study’s primary aim. However, the results of one study (women only [26]) are confounded by differences in total protein intakes between groups. Collectively, the results suggest relatively more balanced protein distributions (CV), when total protein intakes are greater than the protein recommended dietary allowance, are associated with more favorable skeletal muscle-related outcomes.

### 2.2. Number of Meals Reaching a Target Threshold

The previous section compared and contrasted studies that defined protein distribution by the degree of “balancing”. That is, protein distribution was quantified on a continuous scale between 0 and 1 (0 being the most balanced). Another method of exploring the protein distribution concept is to assess whether the number of meals people consume that reach a target MPS threshold result in different muscle-related responses. Hypothetically, persons that consume more meals that elicit maximal MPS responses would have more favorable skeletal muscle-related outcomes (e.g., higher lean mass). In essence, these studies are assessing muscle-related outcomes by moving from a less to more optimal distribution. Two cross-sectional studies by Loenneke et al. [27] and Loprinzi et al. [28] used the same 1999–2002 National Health and Nutrition Examination Survey (NHANES) cohort to investigate the association between the number of meals containing ≥30 g of protein with leg lean mass and knee extensor strength. Participants were stratified into one of three groups: the referent group (0 meals containing ≥30 g protein, *n* = 341); 1 meal containing ≥30 g protein (*n* = 560); 2+ meals containing ≥30 g protein (*n* = 172). Loenneke et al. [27] and Loprinzi et al. [28] both reported that compared to 0 meals, consuming 1 or 2+ meals/d with ≥30 g of protein was associated with greater leg lean mass and knee extensor strength. Loprinzi et al. [28] further investigated whether there was a three-way interplay among moderate to vigorous physical activity and leisure time activities in combination with consuming meals containing ≥30 g of protein. Although activity levels were related to leg lean mass and strength, there was no evidence of a three-way interaction. Gayatán Gonález et al. [29] used the same study design with meal thresholds of ≥30 g and 0.4 g·kg^−1^. Compared to consuming 0 meals, consuming 2 or 3 meals per day, but not 1 meal, with adequate protein content was associated with lower risk of physical disability among 4 (30 g criterion) and 2 (0.4 g·kg^−1^ criterion) of the 15 outcomes assessed. Notably, the reference groups for each study [27,28,29] were consuming less than the recommended dietary allowance for protein. Conversely, the comparator groups (groups consuming 1 and 2+ meals/d above the thresholds) had relative protein intakes greater than 1.0 g·kg^−1^·d^−1^. Although total daily protein quantity was controlled for in the analyses, these results may reflect negative consequences of consuming less than the protein recommended dietary allowance on muscle mass and strength and not the benefits of frequently consuming moderately high-protein-containing meals.

A more recent analysis by Mishra et al. [30] using 2011–2014 NHANES data found that the grip strength of participants consuming ≥25 g of protein in at least one meal was not different compared to those consuming ≥25 g of protein at 2 and 3+ meals/d. These results are contradictory to those reported by Loenneke et al. [27] and Loprinzi et al. [28]. However, they both [27,28] referenced the group consuming 0 meals containing ≥30 g of protein, while Mishra et al. [30] referenced the group consuming at least 1 meal containing ≥25 g of protein. The total protein intakes of the groups consuming at least 1 meal containing ≥25 [30] or ≥30 g [27] of protein were comparable; each consumed 66–76 g·d^−1^ and ~78 g·d^−1^, respectively. It is possible that Mishra et al. [30] would have detected an association had they referenced the group consuming 0 meals that reached the target threshold. However, the low total protein intakes reported in this group would likely confound the results (~40 g·d^−1^); similar to the studies by Loenneke et al. [27] and Loprinzi et al. [28] (~45 g·d^−1^). Conversely, it is possible that Loenneke et al. [27] would not have reported an association had they used the group consuming at least 1 meal/d that reaches the target threshold as the comparator. Mishra et al. [30] also argued that because each study had a different primary outcome (leg lean mass and strength vs. hand grip strength), the conclusions may not be comparable. However, Valenzuela et al. [31], after adjusting for covariates (body weight, sex, and height), found appendicular lean mass was not different among participants from Northwestern Mexico who consumed no meals versus those who consumed at least one meal containing ≥25 g of protein. Similarly, Gingrich et al. [23] also found that the number of meals containing ≥0.4 g·kg^−1^ or ≥2.5 g leucine was not associated with leg strength, leg power, and hand grip strength.

Collectively, three [27,28,29] of six [23,27,28,29,30,31] studies demonstrated that consuming at least one meal that reaches a saturable dose limit is associated with a skeletal muscle-related outcome; however, these three studies [27,28,29] had reference groups consuming less than the protein recommended dietary allowance. Overall, these results indicate that compared to consuming marginal or inadequate protein quantity (<0.8 g·kg^−1^·d^−1^), consuming an unbalanced protein distribution with a higher-protein diet >1.0 g·kg^−1^·d^−1^, where at least one meal contains sufficient protein quantity to maximally stimulate MPS, is helpful to promote skeletal muscle health; consuming meals with “adequate” protein more frequently (i.e., moving from a unbalanced to an “optimal” protein distribution) was not associated with further skeletal muscle-related benefits.

### 2.3. Number of Meals Not Reaching a Target Threshold

In contrast to comparing the skeletal muscle-related outcomes among groups consuming incrementally more meals that reach a target threshold, the protein distribution concept can be approached by assessing the contrapositive: studying the deviation away from an optimal protein distribution. Gayatán Gonález et al. [32] assessed the separate relationships between subjective functionality scores and occasions in which protein intakes were less than either 30 g or 0.4 g·kg^−1^ at breakfast, lunch, or dinner, respectively. That is, they were assessing whether specific meals with “inadequate” protein were associated with functionality: does the meal in which protein “inadequacy” occurs matter? Using the 30 g criterion, inadequate protein intake at lunch was associated with both low and middle scores for activities of daily living. Using the 0.4 g·kg^−1^ criterion, only inadequate protein intake at dinner was associated with middle scores for instrumental activities of daily living. The authors [32] argued this relationship may exist because, among Mexican adults, lunch contributes the most protein towards total daily intakes: low protein intakes at lunch may promote consuming a lower total protein diet (information on total protein intakes were not available). However, even among younger adult Japanese men and women who were consuming greater than the protein recommended dietary allowance, total body fat-free mass percentage was greater in those that consumed ≥0.24 g·kg^−1^ at three meals compared to those that did not consume ≥0.24 g·kg^−1^ in at least one meal [33]. A difference in total protein intakes between groups makes it difficult to determine whether the more favorable fat-free mass status is a result of consuming either a balanced protein distribution or a higher-protein diet. The results from these two studies [32,33] apparently suggest that consuming a protein distribution that is anything less than “optimal” (i.e., where at least one meal contains “inadequate” protein) will be associated with poorer skeletal muscle-related outcomes. This would appear to suggest, based on the methods of the previous Section 2.2, that if the referent group was comprised of persons consuming 2 meals/d that reach a target threshold, then consuming 3 or more meals/day would be associated with more positive muscle-related outcomes. However, this is not readily apparent.

### 2.4. Conclusions from Observational Research

Collectively, the limited inconsistent results among the observational studies suggest that when total protein intakes are above 0.8 g·kg^−1^·d^−1^, consuming a relatively more balanced protein distribution may be superior to an unbalanced protein distribution to promote skeletal muscle-related outcomes. When total protein intakes are less than 0.8 g·kg^−1^·d^−1^ and more balanced, consuming a higher unbalanced protein distribution may be more favorable because at least one meal may contain sufficient protein to support muscle anabolism. However, encouraging people to consume a more balanced distribution may be advised to increase their total protein intakes at or above. 0.8 g/kg.

## 3. Randomized Controlled Research

### 3.1. Acute Protein Ingestion Research

Compared to consuming an unbalanced protein distribution, more proteins may be synthesized by consuming an optimal protein distribution; this seems intuitive: consuming more meals that stimulate a higher MPS rates should result in more synthesized muscle protein. To our knowledge, there are five acute studies that test the within-day protein distribution hypothesis (Table 2) [34,35,36,43,44]. The inaugural study performed in energy balance by Mamerow et al. [34] supports consuming a balanced protein distribution for a 25% higher aggregate MPS over 24 h, although findings from two studies by Kim et al. [35,43] and one by Murphy et al. [36] did not corroborate those results. Two more studies performed while participants were in energy restriction by Murphy et al. [36,44] report conflicting results: one showed that consuming a balanced versus unbalanced protein distribution resulted in ~19% higher MPS [36], while the other failed to reject the null hypothesis [44]. The results of the five acute studies are challenging to aggregate into a single conclusion because of the heterogeneity in participant characteristics, protein sources, and meal protein quantity. Each of these factors could arguably contribute to whether the study resulted in null or differential findings between balanced and unbalanced protein distributions.

Crudely, the protein distribution concept is supported when testing is performed during energy balance in younger [34], but not older [35,36,43], adults. The age of the participants studied is but one factor making it difficult to aggregate the results. Known as anabolic resistance, aging is associated with a disproportionate lesser increase in MPS to increasing intakes of dietary protein [10,45]. This phenomenon was documented in a retrospective analysis by Moore et al. [10]: compared to younger adults, the maximally effective dose of high-quality isolated intact protein to maximize MPS in older adults was ~70% higher. This information was relatively unknown when each of the studies presented here were developed. At the time, the sufficient dose of high-quality protein to maximize MPS, regardless of age, was thought to be 25–40 g [9,46,47,48]. Mamerow et al. [34] provided a 30 g dose of protein, based on their previous research [9], to their balanced group that may have been enough protein at each of the three meals to maximize MPS. Their unbalanced group may have only consumed one meal (dinner) that maximized MPS. In contrast, Kim et al. [35,43] and Murphy et al. [36] likely only provided a balanced protein distribution—not an optimal distribution. These results underscore the importance of tailoring the dose per meal of protein to achieve an optimal protein distribution to the age of the participants.

Apart from age, the inconsistent results could be explained by differences in both protein source and quantity, which consequently impact protein synthesis. In the retrospective analysis by Moore et al. [10], the saturable dose estimates were determined using studies on isolated intact proteins (i.e., protein supplements); these types of protein sources induce both higher peak plasma amino acid concentrations and faster time-to-peak concentration than whole foods [49,50,51]; this could positively impact their availability for influx to skeletal muscle to promote MPS [49,52]. In comparison, the saturable dose estimates were seemingly met by both Mamerow et al. [34] and Kim et al. [35,43] using whole foods. However, Mamerow et al. [34] reported a differential effect of protein distribution on MPS (25% higher in balanced vs. unbalanced) but Kim et al. [35,43] did not. Mamerow et al. [34] provided ~60% more protein than was estimated for their population’s saturable dose [10]. The protein doses used by Kim et al. [35,43] did not exceed the estimates to the same extent for their population. These results suggest that merely meeting a saturable dose estimate at each meal is not adequate. Rather, protein quality is also an important consideration, especially when consuming whole foods with plant and animal sources.

Even when consuming protein supplements, sufficient protein doses still need to be achieved. In energy balance, Murphy et al. [36] provided 25 g of an isolated intact protein—whey protein micellar. This was likely an insufficient dose for this cohort: to maximally stimulate MPS, they likely needed closer to ~40 g of high-quality protein per meal. Failure to either reach the target thresholds with protein supplements or exceed the thresholds with whole foods may explain the discordant results. However, the practicality of having adults consume protein in excess of 40 g in whole foods is up for debate [53].

In energy restriction, Murphy et al. [36,44] presented the only study that assessed the effect of protein distribution with or without resistance training. In their study using a primed, continuous intravenous infusion of an isotopically labelled amino acid, the authors reported that consuming a balanced distribution promoted ~19% higher MPS versus an unbalanced distribution [36]. Why did they find an effect of protein distribution in energy restriction, but not in energy balance? It may be that energy restriction “sensitizes” the muscle to changes in amino acid concentrations [54]; an increase in sensitivity could result in a greater increase between fasting and fed-state MPS at a given protein dose. Indeed, the magnitude of change in MPS from the fasting to fed state, although ultimately depressed with energy restriction, was greater in the balanced group than unbalanced group. However, when Murphy et al. [44] used an oral administration of deuterium oxide to measure bulk myofibrillar protein synthesis rates over two weeks of energy restriction, the results were not replicated. The authors speculated on several reasons for this. (1) Short-term laboratory measurements of MPS are sensitive to measuring acute responses to either feeding or exercise stimuli. Longer measures of MPS would inherently reflect variations in daily exposure to stressors. (2) The first study [36] was performed in the postprandial state only; the second study [44] included fasting rates in the calculations of fractional synthetic rates; this could have “diluted” the protein synthesis rates and masked the effect of protein distribution. (3) The first study included measurements of MPS in response to consuming three liquid meals, each containing 25 g of protein. The second study included participants consuming 1.3 g·kg^−1^·d^−1^ over four whole-food meals. As mentioned above, a blunted rise in postprandial plasma amino acid concentration from consuming whole-food protein sources, although protein intake was higher per meal, could have led to the discrepant results. Again, a higher protein dose may be needed with whole foods than with protein supplements.

The findings from Kim et al. [35,43] and Murphy et al. [36,44], although possibly statistically underpowered, are equally as intriguing as those presented by Mamerow et al. [34]. Despite reporting null results, their results suggest consuming an unbalanced protein distribution, where we assume at least one meal maximizes MPS, is comparable to consuming a balanced protein distribution that fails to reach the “target threshold” at each meal at intakes at and above the protein recommended dietary allowance.

#### 3.1.1. Future Research Directions for Acute Protein Ingestion Research

While it is known that the quantity and quality of the protein consumed influences MPS, we still do not know the contribution of each individual postprandial MPS response to the daily synthesized protein pool. There are many stimuli that can affect MPS; under the most favorable feeding conditions, it is likely the anabolic effect of feeding only partially contributes to total daily protein synthesis. In laboratory settings, we assume the aggregate MPS response over the measurement period results from the sum of the individual postprandial MPS responses. The research by Mamerow et al. [34] suggest that altering the protein content within meals can impact MPS by a substantial margin in the laboratory, while the research by Murphy et al. [44] suggests this may not be the case in a free-living, community setting. This needs to be sufficiently reproduced before definitive conclusions can be drawn.

When performing these types of acute studies without titration, we do not know definitively when an individual or group of participants have maximized their ability to synthesize proteins; we only assume based on previous research under similar conditions. Consequently, we make evidenced-based assumptions when meals likely did not provide enough protein to meet or exceed the saturable dose estimates; often, this occurs as an explanation for why consuming a balanced protein distribution did not result in higher MPS rates. In practical terms, real-world meals typically contain a variety of whole-food animal- and plant-protein sources. While we have reasonable saturable dose estimates for isolated protein sources, future research should address how much protein is needed when consumed in a mixed meal with whole-food plant and animal protein sources. We could then design more ecologically valid studies to test whether an “optimal” protein distribution exists. By extension, we need studies in both younger and older cohorts; they should be designed to detect the potential for sexual dimorphisms; they should be tested in a range of energy balance states. Of course, while testing each of these factors in a single study would be ideal, it is likely not practical. With this in mind, we should strive for designing multiple studies with similar criteria to address these questions.

#### 3.1.2. Conclusions from the Acute Protein Ingestion Research

When we consider the protein quantities within meals relative to the age of the participants within each study, it is clear that a cursory tallying of the results is not sufficient. Hindering a uniform conclusion, we have several studies reporting a null effect [35,36,43] pitted against the strongest evidence from a single study [34] supportive of the “optimal” protein distribution concept. Collectively, at present, convincing evidence is lacking from acute protein ingestion research to support or refute the hypothesis that optimizing protein intake among multiple daily main meals enhances MPS.

### 3.2. Chronic Protein Ingestion Research

The notion that the time of day, frequency, and distribution of daily dietary protein intake could influence protein metabolism dates back to the late 1930s. Researchers at the time manipulated dietary protein consumption frequency and distribution to effect (1) protein/amino acid balance and (2) nitrogen balance. These two balances are thought to be connected through the free amino acid pool. Constituting only a fraction of the total nitrogen pool, the free amino acid pool is maintained within narrow limits; therefore, when the body is in nitrogen balance (input = output), then we presume protein synthesis and breakdown are in balance [2]. However, nitrogen balance studies have their limitations [55,56,57,58,59,60]. With the advent of stable isotope amino acid methodologies, we can more accurately track the flux of amino acids in and out of tissues. Accordingly, the flux of amino acids from skeletal muscle and their incorporation into muscular proteins were emphasized in the 2000s to quantify protein synthetic rates in humans. Evidence from acute stable isotope studies provided cursory support for the within-day day protein distribution to impact skeletal muscle mass and function [34,35,36]. This prompted more research using prospective randomized controlled trial designs in which the relationship between protein distribution and skeletal muscle-related outcomes beyond acute changes in MPS could be further elucidated. This section will provide an in-depth analysis of the more valid nitrogen balance studies and of the more recent longitudinal randomized controlled trials on whole-body composition outcomes.

#### 3.2.1. Nitrogen Balance

For most the 20th century, highly controlled nitrogen balance studies were considered the gold standard for studying protein metabolism; in fact, results from nitrogen balance studies still inform adult protein requirements [1]. Although recent protein metabolism research has emphasized the protein distribution concept, it was of scientific interest as early as 1939. The first study, to our knowledge, was performed using a single male participant by Cuthbertson and Munro [61]. Their initial research was focused on understanding the relationship between carbohydrate and protein metabolism; this inevitably led them to studying the effect of the within-day protein intake frequency—a form of protein distribution. Since their initial research, the effect of protein distribution on nitrogen balance has been studied at least 11 more times [7,38,39,61,62,63,64,65,66,67,68,69]. Many of these studies, however, do not sufficiently meet the criteria for a valid nitrogen balance experiment [70] (e.g., appropriate stabilization periods; periods consuming the controlled diets must be long enough; corrections for integumental and miscellaneous losses; urine and fecal collections must be precisely timed and complete). Considering the evidence in light of these criteria, we considered only two of the 12 studies—one study shows an effect of protein distribution [38] and the other shows that protein distribution does not influence nitrogen balance [39,62] (Table 3).

Arnal et al. [38] were the first to study protein distribution in older women. Participants were randomized to consume either 80% of their protein at lunch (unbalanced) or a more balanced protein distribution pattern (21:31:19:28% protein/meal). After 14 d, the unbalanced group had a more positive nitrogen balance and a maintenance of fat-free mass; fat-free mass slightly decreased (~−0.3 kg) in the balanced group. This was attributed to the greater 24 h whole-body protein synthesis in the unbalanced group than the balanced group, although fed-state protein synthesis rates were not different. The same study design was replicated in younger women by the same group [39]. However, this time the authors reported no difference between groups in whole-body fat-free mass, protein turnover, and protein synthesis and breakdown. While there was no statistically supported difference in whole-body nitrogen balance, nitrogen retention was 1.5 times higher with the balanced vs unbalanced distribution (*p* = 0.16). The authors highlight the difference in nitrogen balance between groups was 23 mg N·kg fat-free mass^−1^·d^−1^ (balanced > unbalanced); this was similar in magnitude to the difference reported in the older females (27 mg N·kg fat-free mass^−1^·d^−1^; balanced < unbalanced) [38,39].

These results from Arnal et al. [38,39] are consistent with an anabolic resistance to feeding commonly observed among older adults. Although four balanced meals were consumed, the quantity of protein at each meal may have been too low to maximally, or at least meaningfully, stimulate MPS [38], i.e., they consumed a balanced but not optimal protein distribution. Conversely, while consuming the unbalanced protein distribution, participants likely maximized MPS after the lunch meal. Even if MPS after lunch was not maximized, these results that suggest aggregate protein synthesis was higher after consuming an unbalanced distribution pattern that likely maximized MPS at least once. Among younger adults who are more “sensitive” to dietary protein/amino acid intake, the lower protein doses consumed in a balanced protein distribution likely triggered a more robust anabolic response to feeding [39]. The postprandial MPS responses while consuming the unbalanced distribution likely were not greatly affected; the maximum achievable MPS rate is not different between younger and older adults [71]; therefore, the MPS responses between groups after lunch were likely comparable.

#### 3.2.2. Body Composition

Acute human clinical studies often provide the foundational science used to design longitudinal studies. Longitudinal randomized controlled trials have the benefit of inherently capturing daily fluctuations in hormonal concentrations, energy intakes, and exposure to stress; these factors can have profound impacts on both phenotypical and functional outcomes. Chronic feeding studies are necessary to assess the applicability and efficacy of acute studies in real-world settings. Only five longitudinal studies to date (to our knowledge) have investigated the effect of protein distribution on body composition [43,62,72,73,74]. One study supports consuming a balanced protein distribution [73], three report null results [43,62,74], and one supports consuming an unbalanced distribution for lean mass gains [72] (Table 4).

Among all randomized controlled trials, only one study supports consuming a balanced protein distribution to support lean mass gains [73]. In this study, Yasuda et al. [73] tested the effect of providing participants with either a low or high-protein breakfast. The high-protein breakfast was achieved by the addition of a protein supplement; the same supplement was consumed at dinner by the low-protein breakfast group. Both groups experienced resistance-training induced increases in lean body mass over time; however, the increases tended to be greater in the high-protein breakfast group (2.5 ± 0.3 kg vs. 1.8 ± 0.3 kg; *p* = 0.056); there was no effect on appendicular lean mass changes. Notably, the balanced group (high-protein breakfast) had a lower total protein intake (1.3 g·kg^−1^·d^−1^) than the unbalanced group (lower-protein breakfast; g·kg^−1^·d^−1^). However, due to small samples sizes in both groups, albeit a large effect size (*d* = 0.7), this study is likely more proof of concept. Only one other study [74] seemingly provided enough protein per meal to be considered an “optimal” distribution pattern; however, contrary to Yasuda et al. [73], Hudson et al. [74] did not report an effect of protein distribution on lean mass changes. Based on the research by Mamerow et al. [34], Hudson et al. [74] prescribed 30 g of protein per meal (~0.3 g·kg^−1^·meal^−1^); this should have provided a “safety margin” of ~0.1 g·kg^−1^ [10]. As mentioned in the acute protein ingestion section above, their estimates are based on studies utilizing isolated intact proteins [10]. Mixed-nutrient meals, analogous to the one meal by Hudson et al. [74], contain multiple whole-food protein sources; the variability in protein quality and the difference in food matrices alters the protein digestion and amino acid absorption kinetics compared with protein supplements [49]. Underscoring this point, Kim et al. [43] found that peak plasma leucine concentrations after consuming a whole-food meal were less than 50% of the peak concentration achieved from consuming an essential amino acids mixture containing twice the leucine quantity. Compared to a supplement, the protein quantity within mixed-nutrient meals likely needs to be greater. Yasuda et al. [73] prescribed a protein supplement, in addition to the standardized whole-food breakfast, to attain the higher-protein meal. Sufficient quantity (0.33 g·kg^−1^) and quality (whole foods and supplement) may have been adequate to achieve a saturable dose and promote an optimal distribution. Although Hudson et al. [74] prescribed a balanced protein distribution, they may not have prescribed an “optimal” protein distribution due to the quality of protein consumed in the mixed meals. Conversely, the unbalanced protein distribution may have provided the protein dose required to stimulate MPS maximally after dinner; the result may have been comparable daily synthesis rates. The same reasoning could be used to explain the null results among the two other studies [43,62]: they prescribed balanced but not “optimal” protein distribution patterns. These arguments, however, are only conjecture; none of the studies measured postprandial MPS rates.

The research by Bouillanne et al. [72] in hospitalized older adults showed that consuming an unbalanced protein distribution supported lean mass gains, while consuming a balanced protein distribution resulted in a loss of lean mass. Similarly, Hudson et al. [74] found younger adults in energy restriction trended (*p* = 0.067) towards losing less lean mass when they consumed an unbalanced protein distribution (−0.5 kg) versus consuming a balanced protein distribution (−1.5 kg). These results [72,74] seemingly agree with the observational results from Loenneke et al. [27] and Loprinzi et al. [28] and the nitrogen balance data provided by Arnal et al. [38]: consuming ≥1 meal with sufficient protein to theoretically maximize MPS may be better for lean body mass retention than consuming three balanced meals with “insufficient” protein to be considered optimal.

While protein quantity within meals may partially explain the null results, the interplay between the anticipated effect size and the studies durations could provide further context. Per annuum, lean mass decreases by approximately 1%–2% on average in adults who typically consume an unbalanced protein distribution [75,76]. We assume the “best-case scenario” for consuming a balanced protein distribution in adults not purposefully altering their body composition over the year would be a retention of lean mass. The amount of lean mass possibly retained over 12 months, being relatively small, is difficult to detect with current techniques. Measuring a differential change between groups who retained and who lost lean mass over 2–4 months, the length of the studies presented here, may be even less feasible. Aligning with this, Kim et al. [43] hypothesized that consuming a balanced vs unbalanced protein distribution over 8 weeks would increase lean mass [77]; however, they showed that lean mass did not differentially change. Even with weight loss interventions, where lean mass loss is expected, both Adechian et al. [62] and Hudson et al. [74] reported that lean mass did not differentially change between groups who were energy-restricted even though the interventions were longer. Bouillanne et al. [72] reported differential lean mass changes after only 6 wk; however, they studied a hospitalized, malnourished population. Among a population experiencing lean mass loss above the 1%–2% rate, an effect of protein distribution may be measurable with our current techniques under “shorter” durations.

An exercise stressor has been proposed to provide the “optimal environment” for protein distribution to induce differential changes in lean mass, including skeletal muscle, over a few months. Compared to protein ingestion while being sedentary, resistance training “sensitizes” the muscle to promote greater MPS [47]. Theoretically, consuming a balanced distribution of protein among daily meals would stimulate MPS more frequently, in turn enhancing resistance training-induced lean mass and muscle accretion [44]. An alternative hypothesis is that manipulating dietary protein distribution concurrently with resistance training would not influence lean mass and muscle accretion because the anabolic effect of resistance training is superior to the relatively less robust effect of protein distribution [78]. No study to date has directly tested these opposing hypotheses regarding body composition.

As discussed, postprandial MPS partly determines changes in the skeletal muscle protein pool; however, we must acknowledge that both acute and chronic studies have several limitations. First, skeletal muscle changes in response to repeated exposures to both catabolic and anabolic stressors. These stressors include, but are not limited to, hormonal concentrations, energy status, and exercise training status. Their influence on lean mass changes cannot be accurately predicted from acute studies because they fluctuate and persist beyond the durations measured in laboratory settings. Second, the saturable dose estimates were established by sampling a single muscle—the *vastus lateralis*. While MPS does not vary by either anatomic location or fiber type [79], lean mass is not solely composed of skeletal muscle. Changes in lean mass also reflect changes in organ tissues and water. Third, hydration status can have a particularly profound influence on lean mass because water fluctuates to a greater magnitude than the muscle protein pool. In an attempt to improve the congruence between lean mass and skeletal muscle, a four-compartment model of body composition was developed to factor-in fluctuation in total body water [80,81]. This would get us closer to measuring changes in the protein pool alone. Another viable alternative to estimating changes in the protein content of skeletal muscle is to measure appendicular lean mass. The appendicular regions have more congruence with skeletal muscle than whole-body lean mass for the following: it is devoid of organs, is less prone to major fluctuations in body water, and is comprised primarily of skeletal muscle. However, surrogate markers of the skeletal muscle protein pool should always be interpreted with caution.

#### 3.2.3. Future Research Directions for Chronic Protein Ingestion Research

We argued that the lack of evidence in support of an optimal protein distribution may be explained by both inadequate protein quantity and quality within meals; this resulted in balanced but not optimal protein distributions. The saturable dose estimates we used to critique meal protein quantity are based on isolated intact proteins. However, importantly, the vast majority of protein consumed comes from protein-rich foods, not supplements. Establishing the relative saturable doses using whole-food animal- and plant-protein sources will facilitate conducting optimal distribution studies while participants consume varied protein-rich foods.

Recommendations in the scientific literature are made for older adults to consume 0.4–0.6 g·kg^−1^·meal^−1^ to promote muscle retention [82]. This equates to 1.2–1.8 g·kg^−1^·d^−1^, which is 50% to 125% higher than the recommended dietary allowance and ~15% to 110% higher than the average daily protein intake amongst older adult men and women [19]. Currently, there is no direct evidence from randomized controlled trials to support consuming 1.2–1.8 g·kg^−1^·d^−1^ in a balanced protein distribution to promote muscle size, strength or quality. There is evidence these quantities of protein intake do promote beneficial changes in lean mass; however, this is compared to consuming lower-protein diets [83,84,85,86]. In these studies, the higher protein intakes may have been achieved by pragmatically adding the additional protein to breakfast and lunch; the dinner meal may not have been a good candidate for additional protein because it is usually the largest protein-containing meal. Therefore, it is possible we have evidence that a balanced higher-protein diet is more beneficial for lean mass than consuming an unbalanced lower/normal protein diet. We currently lack sufficient evidence to determine the effect of protein distribution on lean mass when total protein intake is matched at either lower or higher protein intakes.

Arguably, research designed to measure muscular function outcomes may be more meaningful than those measuring lean mass. Skeletal muscle strength and function, rather than mass, is often associated with health and longevity among aging adults [87]. Shifting our research focus away from measuring mass and towards assessing function may be the future of protein ingestion research.

#### 3.2.4. Conclusions from Chronic Protein Ingestion Research

While balanced versus unbalanced protein distributions have been experimentally tested, there is likely only one study assessing an “optimal” protein distribution [73]. The distinction between balanced and “optimal” protein distribution is an important one. Collectively, the results suggest that consuming an unbalanced protein distribution with at least one high-protein meal/d may be equally or more beneficial than a balanced, but not optimal, protein distribution on lean mass. Consuming a diet with both a balanced and optimal protein distribution pattern may confer advantages over an unbalanced distribution on lean mass gains.

## 4. Conclusions—Does the Evidence Support the Concept?

There seems to be a valid theoretical rationale to optimize protein distribution to influence muscle-related outcomes. However, the current available evidence is too limited and inconsistent to make a definitive conclusion about whether changing dietary patterns from consuming an unbalanced distribution to consuming an “optimal” protein distribution pattern will positively influence muscle-related outcomes. The underlying rationale for promoting an optimal protein distribution throughout the day remains intriguing but, from the available literature, it appears more important to ensure adequate total daily protein intake. Because typical protein distribution patterns are skewed towards the dinner meal, encouraging adults, especially older adults with marginal or inadequate protein intakes (<0.80 g·kg^−1^·d^−1^), to better balance their daily protein intake, by consuming more protein at breakfast and lunch meals, may be a practical way to achieve a moderately higher total protein diet and promote skeletal muscle health. However, recommending individuals who consume a low-protein diet to balance protein distribution without increasing their total protein intake to become adequate is ill-advised. Among individuals who consume adequate total protein (0.8–1.3 g·kg^−1^·d^−1^), the preponderance of evidence suggests that consuming at least one high-protein meal per day may be sufficient to support skeletal muscle-related outcomes even if the distribution is unbalanced. 

## Figures and Tables

**Table 1:** Observational study characteristics.

Authors	Population	Techniques	Outcome
Bollwein et al. [22]	194 adults from Nurnberg, Germany (≥75 y)	Dietary intake: food frequency questionnaire. Frailty: definition by Fried et al. [41]	Non-frail participants reported a more balanced protein distribution [median CV (min–max); 0.68 au (0.15–1.24)] than pre-frail [0.74 (0.07–1.29)] and frail participants [0.76 (0.18–1.33); total protein intakes were comparable (~1.1 g·kg^−1^·d^−1^)
Gingrich et al. [23]	97 adults without functional limitations from Nuremburg, Germany (75–85 y)	Dietary intake: 7-d food records. Body composition: BIA. Skeletal muscle mass: estimated [42]. Leg strength, leg power, and hand grip strength: dynamometers	No association between daily protein intake quantity, balance of within-day distribution (CV), number of meals containing ≥0.4 g·kg^−1^, and number of meals containing ≥2.5 g leucine with leg strength, leg power, and hand grip strength
Ten Haaf et al. [24]	140 community-dwelling adults from the Netherlands (81 ± 6 y)	Dietary intake: average of 2, 24-h recalls or from 3-d food records. Hand grip strength: dynamometer. Physical function: Short Physical Performance Battery tests. Quality of life: EQ-5D-5L.	Among the five outcomes, a more balanced protein distribution (spread CV < 0.43) was associated only with greater gait speed (β = −0.42) when compared to the intermediate CV (0.43–0.62)
Farsijani et al. [25]	2-y follow up in 351 men and 361 women from the Quebec Longitudinal Study on Nutrition as a Determinant of Successful Aging study (67–84 y)	Dietary intake: average of 3, 24-h food recalls collected at baseline and at 2 y. Body composition: DXA	At baseline, men with the most balanced protein distribution (CV ≤ 0.33 au) had higher whole-body and appendicular lean mass at baseline than did those with the most unbalanced distribution (CV ≥ 0.67 au). No differences among women. At 2-y, a more balanced distribution was negatively associated with higher whole-body and appendicular lean mass in both men and women. Protein distribution was not associated with changes in lean tissue over 2 y
Farsijani et al. [26]	3-y follow up in 827 men and 914 women from the Quebec Longitudinal Study on Nutrition as a Determinant of Successful Aging study (67–84 y)	Dietary intake: data from the 2-y follow-up. Hand, leg, and arm strength: dynamometers. Mobility: timed-up-and-go, chair stand, and walking speed tests	A more balanced distribution was associated with a higher muscle strength score at 2-y in men and women (β ± SE = −0.73 6 ± 0.20 and −0.66 ± 0.20, respectively). Similar negative associations were observed between protein distribution with handgrip and arm strengths. These associations were significant before and after adjustment for covariates in women and only before adjustment for covariates in men with a trend toward significance after adjustment. The association between leg strength and protein distribution was not significant in either sex. Protein distribution was not associated with the decline in composite and component mobility scores
Loenneke et al. [27] and Loprinzi et al. [28]	1081 adults from the 1999–2002 NHANES cohort of (50–85 y)	Dietary intake: a 24-h dietary recall method. Leg lean mass: DXA. Knee extensor strength: dynamometer	Compared to 0 meals, consuming 1 and 2+ meals/d with ≥30 g of protein was associated with greater leg lean mass (1 vs. 0, β = 23.6; 2+ vs. 0, β = 51.1) and knee extensor strength (1 vs. 0, β = 1160; 2+ vs. 0, β = 2389)
Gayatán Gonález et al. [29]	187 adults from Mexico (60–97 y)	Dietary intake: a 24-h dietary recall method on a single day. Functionality: questionnaire to determine ADL and IADL scores	Compared to 0 meals, consuming 2 or 3, but not 1, meals/d with ≥30 g protein was associated with lower risk of physical disability on transportation (OR [95% CI]: 0.06 [0.01–0.50]), shopping (0.05[0.01–0.40]), feeding (0.06 [0.01–0.74]), and transfer (0.09 [0.01–0.98]). Consuming 2 or 3, but not 1, meals/d with ≥0.4 g/kg was associated with lower risk of physical disability on shopping (0.21 [0.05–0.89]) and transportation (0.12 [0.03–0.48])
Mishra et al. [30]	4123 adults from the 2011–2014 NHANES cohort (≥51 y)	Dietary intake: a 24-h dietary recall method. Grip strength: hand dynamometer	Compared to 1 meal, consuming 2 and ≥3 meals containing ≥ 25 g protein was not associated with grip strength
Valenzuela et al. [31]	78 adults from Mexico (68.7 ± 6.3 y)	Dietary intake: a 24-h dietary recall method on 3 non-consecutive days. Appendicular lean mass: DXA	After adjusting for weight, sex, and height, appendicular lean mass was not different between groups that consumed at least one meal containing ≥25 g of protein and those who did not
Gayatán Gonález et al. [32]	190 adults from Mexico (53–97 y)	Dietary intake: a 24-h dietary recall method on a single day. Functionality: questionnaire to determine ADL and IADL	30 g criterion: Low and middle ADL scores were associated with “inadequate” protein intake at lunch (low scores, OR = 3.82 [95% CI, 1.15–12.65]; middle scores, OR = 2.40 [1.03–5.62]). 0.4 g·kg^−1^ criterion: “Inadequate” protein intake at dinner was associated with middle IADL scores (OR = 7.64, [1.27–45.85])
Yasuda et al. [33]	233 adults from Japan (21.4 ± 2.4 y)	Dietary intake: photography on 3 non-consecutive days. Body composition: DXA	Total fat-free mass % was greater in those that consumed ≥0.24 g·kg^−1^ at three meals compared to those that did not consume ≥0.24 g·kg^−1^ in at least one meal (77.0 ± 0.5% vs. 75.2 ± 0.4%)

Abbreviations: ADL, activities of daily living; BIA, bioelectrical impedance analysis; DXA, dual-energy-ray absorptiometry; CI; confidence interval; CV, coefficient of variation; IADL, instrumental activities of daily living; NHANES, National Health and Nutrition Examination Survey; OR, odds ratio; SE, standard error.

**Table 2:** Acute randomized controlled protein ingestion studies.

										Protein, g (g/kg)					
Authors	Study Design	Group ^1^	*n* (f)	Age, y	Duration	Energy Status	Exercise Status	Meal Type	Protein Source (s)	Total	Breakfast	Lunch	Dinner	4th Meal	Outcome
Mamerow et al. [34]	Cross-over	EVEN	8	36.9 ± 3.1	24 h	EB	No RT	Whole-food	Animal and plant	90 (1.17)	30 (0.39)	30 (0.39)	30 (0.39)		25% greater MPS in EVEN
		SKEW								90 (1.17)	10 (0.13)	15 (0.20)	65 (0.85)		
Murphy et al. [36]	Cross-over within parallel	BAL	10	65 ± 3	12 h	EB and ER phases	RT and No RT phases	Isolated intact protein	Whey micellar	75 (0.77)	25 (0.26)	25 (0.26)	25 (0.26)		19% greater MPS in BAL in ER and ER + RT; no effect in EB
		SKEW	10	66 ± 4						75 (0.78)	10 (0.10)	15 (0.16)	50 (0.52)		
Kim et al. [35]	Parallel	RDA Even	5 (4)	66.4 ± 1.7	22 h	EB	No RT	Whole-food	Animal and plant	65.8 (0.8)	22.3 (0.3)	21.5 (0.2)	22 (0.3)		No effect
		RDA Uneven	4 (1)	64.0 ± 3.6						73.7 (0.8)	11.1 (0.1)	14.9 (0.2)	47.8 (0.5)		
		2RDA Even	5 (3)	64.0 ± 2.7						112.4 (1.5)	38 (0.5)	36.5 (0.5)	37.9 (0.5)		
		2RDA Uneven	6 (2)	68.4 ± 2.2						120.8 (1.4)	18.1 (0.2)	24.3 (0.3)	78.4 (0.9)		
Kim et al. [43]	Parallel	Even	7 (3)	58.1 ± 2.4	23 h	EB	No RT	Whole-food	Animal and plant	(1.1)	(0.37)	(0.37)	(0.37)		No effect
		Uneven	7 (5)	60.3 ± 2.4						(1.1)	(0.17)	(0.22)	(0.72)		
Murphy et al. [44]	Parallel	BAL	10	66 ± 4	2 wk	ER	RT and No RT phases	Whole-food	Animal and plant	(1.3)	(0.33)	(0.33)	(0.33)	(0.33)	No effect
		SKEW	10							(1.3)	(0.09)	(0.22)	(0.94)	(0.05)	

^1^ Group names reflect designations by the study authors. Abbreviations: EB, energy balance; ER, energy restriction; MPS, muscle protein synthesis; RDA, recommended dietary allowance; RT, resistance training.

**Table 3:** Chronic protein ingestion research assessing the effect of protein distribution on nitrogen balance.

Authors	*n* (f)	Age, y	Protein, g·kg^−1^·d^−1^	Meals, Number/d (g/kg/Meal)	Protein Sources	Adaptation/ Collection, d	Results
Arnal et al. [38]	15	68 ± 1	1.05	3 (0.1/0.8/0.15) vs. 4 (0.22/0.33/0.2/0.3)	Whole-food animal and plant	15/14	Nitrogen balance was higher in 3 meals (unbalanced, 54 ± 7 mg/fat-free mass) vs. 4 meals (balanced, 27 ± 6 mg/fat-free mass)
Arnal et al. [39]	16	26 ± 1	1.2	3 (0.08/0.95/0.17) vs. 4 (0.26/0.37/0.23/0.34)	Whole-food animal and plant	15/14	Nitrogen balance was 60% lower in 3 (unbalanced, 36 ± 8 mg/fat-free mass) vs. 4 meals (balanced, 59 ± 12 mg/fat-free mass) (*p* = 0.16)

**Table 4:** Chronic protein ingestion research assessing the effect of protein distribution on body composition.

Authors	Group ^1^	*n* (f)	Age, y	Duration	Energy Status	Exercise Status	Protein Source(s)	Protein, g (g/kg)					Results
								Total	Breakfast	Lunch	Snack	Dinner	
Bouillanne et al. [72]	Spread	34 (23)	85.7 (83.5–87.9)	6 wk	None	None	Animal and plant	69 (1.27)	12.2 (0.25)	21 (0.38)	13.5 (0.25)	21.1 (0.38)	Pulse feeding increased lean mass (0.91 [0–1.48]); spread feeding decreased lean mass (−0.41 [1.53–0.49])
	Pulse	29 (23)	84.1 (81.8–86.4)					66 (1.31)	4.5 (0.08)	47.8 (1.02)	2.3 (0.03)	10.9 (0.14)	
Kim et al. [43]	EVEN	7 (3)	58.1 ± 2.4	8 wk	EB	No RT	Animal and plant	87.8 (1.1)	29.3 (0.37)	29.3 (0.37)	—	29.2 (0.37)	No effect
	UNEVEN	7 (5)	60.3 ± 2.4					86.4 (1.1)	13.1 (0.16)	17.7 (0.22)	—	55.6 (0.7)	
Adechian et al. [62]	Casein spread	10 (8)	35.1 ± 1.5	6 wk	ER	None	>80% casein	87 (0.94)	22 (0.24)	22 (0.24)	22 (0.24)	22 (0.24)	No effect
	Casein pulse	10 (8)	34.6 ± 1.4				>80% casein	87 (0.96)	7 (0.08)	70 (0.77)	3 (0.04)	7 (0.08)	
	MSP spread	11 (7)	33.6 ± 1.8				>80% MSP	87 (0.93)	22 (0.23)	22 (0.23)	22 (0.23	22 (0.23	
	MSP pulse	10 (9)	30.6 ± 2.3				>80% MSP	87 (1.01)	7 (0.09)	70 (0.80)	3 (0.04)	7 (0.09)	
Hudson et al. [74]	EVEN	21	33	16 wk	ER	RT	70% animal; 30% plant	90 (1.1)	30 (0.36)	30 (0.36)	—	30 (0.36)	No effect
	SKEW	20	36					90 (1.1)	10 (0.12)	20 (0.24)	—	60 (0.71)	
Yasuda [73]	Low-protein breakfast	14	20.8 ± 0.4	12 wk	None	RT	Animal, plant, and supplement	97.1 (1.45)	7.7 (0.12)	30 (0.45)	—	55.4 (0.83)	Lean mass increases tended to be greater after consuming the high-protein breakfast (2.5 ± 0.3 kg) than after consuming the low-protein breakfast (1.8 ± 0.3 kg) (*p* = 0.06)
	High-protein breakfast	12						89.4 (1.3)	22.6 (0.33)	31.8 (0.46)	—	32.4 (0.48)	

^1^ Group names reflect designations by the study authors. Abbreviations: EB, energy balance; ER, energy restriction; MSP, milk-soluble protein; RT, resistance training.
